# Acute sleep deprivation in mice generates protein pathology consistent with neurodegenerative diseases

**DOI:** 10.3389/fnins.2024.1436966

**Published:** 2024-07-24

**Authors:** Rachel K. Rowe, Philip Schulz, Ping He, Grant S. Mannino, Mark R. Opp, Michael R. Sierks

**Affiliations:** ^1^Department of Integrative Physiology, University of Colorado Boulder, Boulder, CO, United States; ^2^Chemical Engineering, The School for Engineering of Matter, Transport and Energy, Arizona State University, Tempe, AZ, United States

**Keywords:** sleep, sleep disruption, neurodegeneration, Alzheimer’s disease, pathology

## Abstract

**Introduction:**

Insufficient or disturbed sleep is strongly associated with adverse health conditions, including various neurodegenerative disorders. While the relationship between sleep and neurodegenerative disease is likely bidirectional, sleep disturbances often predate the onset of other hallmark clinical symptoms. Neuronal waste clearance is significantly more efficient during sleep; thus, disturbed sleep may lead to the accumulation of neuronal proteins that underlie neurodegenerative diseases. Key pathological features of neurodegenerative diseases include an accumulation of misfolded or misprocessed variants of amyloid beta (Aβ), tau, alpha synuclein (α-syn), and TarDNA binding protein 43 (TDP-43). While the presence of fibrillar protein aggregates of these neuronal proteins are characteristic of neurodegenerative diseases, the presence of small soluble toxic oligomeric variants of these different proteins likely precedes the formation of the hallmark aggregates.

**Methods:**

We hypothesized that sleep deprivation would lead to accumulation of toxic oligomeric variants of Aβ, tau, α-syn, and TDP-43 in brain tissue of wild-type mice. Adult mice were subjected to 6 h of sleep deprivation (zeitgeber 0–6) for 5 consecutive days or were left undisturbed as controls. Following sleep deprivation, brains were collected, and protein pathology was assessed in multiple brain regions using an immunostain panel of reagents selectively targeting neurodegenerative disease-related variants of Aβ, tau, α-syn, and TDP-43.

**Results:**

Overall, sleep deprivation elevated levels of all protein variants in at least one of the brain regions of interest. The reagent PDTDP, targeting a TDP-43 variant present in Parkinson’s disease, was elevated throughout the brain. The cortex, caudoputamen, and corpus callosum brain regions showed the highest accumulation of pathology following sleep deprivation.

**Discussion:**

These data provide a direct mechanistic link between sleep deprivation, and the hallmark protein pathologies of neurodegenerative diseases, such as Alzheimer’s and Parkinson’s diseases.

## 1 Introduction

It is estimated that more than 50 million American adults suffer from at least one sleep disorder, and up to one third of the United States population reports regularly getting insufficient sleep (Colten and Altevogt, 2006; Paruthi et al., 2016). Insufficient or disturbed sleep is strongly associated with a vast array of adverse health conditions, including various neurodegenerative disorders (Guarnieri et al., 2012; Tsapanou et al., 2015; Vanderheyden et al., 2018; Leng et al., 2019; Minakawa et al., 2019). While the relationship between sleep and neurodegenerative diseases is likely bidirectional, sleep disturbances often predate the onset of cardinal diagnostic clinical symptoms (Abbott and Videnovic, 2016). This recurrence posits sleep as a key mediator of metabolic and inflammatory processes known to affect both disease onset and progression (Green et al., 2020; Reddy and van der Werf, 2020).

During slow-wave sleep, drainage of interstitial waste products and soluble proteins occurs in the brain via fluid interchange between the interstitial space and perivascular space in an arrangement known as the glymphatic system (Xie et al., 2013; Jessen et al., 2015). During sleep, interstitial volume increases, leading to more availability of interstitial fluid (ISF) and cerebrospinal fluid (CSF), and less resistance to their convective fluxes (Xie et al., 2013; Musiek and Holtzman, 2016). Thus, neuronal waste clearance is significantly more efficient during sleep. In opposition to the hypothesis that sleep promotes the clearance of toxins from the brain, a recent study has shown that brain clearance of a small dye that can freely move in extracellular space is reduced during sleep and anesthesia (Miao et al., 2024). This necessitates more research to uncover the mechanism of how toxins are cleared from the brain and determine if molecules of larger molecular weights move differently during sleep-related glymphatic clearance (Miao et al., 2024).

Wakefulness, on the other hand, increases sympathetic output and suppresses glymphatic system function (Musiek and Holtzman, 2016). Even a single sleepless night reduces amyloid beta (Aβ) clearance, a pathological hallmark of Alzheimer’s disease (AD), in the brains of healthy adults (Ooms et al., 2014; Shokri-Kojori et al., 2018). Moreover, sleep deprivation also increases CSF markers of neuronal injury and can result in the dysregulation of pro-inflammatory cytokines known to potentiate the pathogenesis of AD (Musiek and Holtzman, 2016; Green et al., 2020). In addition to changes in Aβ glymphatic clearance, the resulting cellular stress induced by sleep loss is also associated with mitochondrial stress which triggers apoptotic pathways propagating cell death (Williams and Naidoo, 2020). Beyond cellular stress, sleep loss is associated with additional routes of proteostasis failure through impairments of proteosome, autophagy, and unfolded protein response (Williams and Naidoo, 2020; Morrone et al., 2023). This phenomenon extends well beyond Aβ and AD, with sleep loss affecting the homeostasis of neuronal protein variants that underly many neurodegenerative diseases (Owen and Veasey, 2020; Morrone et al., 2023).

While sleep loss can lead to cellular stress at a global level, imaging studies suggest that specific brain regions may be differentially altered by sleep loss. For example, following sleep deprivation, patients show reductions in functional MRI (fMRI) signals while performing attention tasks, specifically in the dorsolateral prefrontal cortex and intraparietal sulcus (Chee and Tan, 2010; Chee et al., 2011). Other studies report differential alterations in function across multiple brain regions and networks after sleep loss, including the thalamus, medial prefrontal cortex, and posterior cingulate cortex (Krause et al., 2017). Beyond functional alterations, sleep loss can cause volumetric and neuronal loss throughout the brain, and these reductions do not fully reverse with recovery of sleep (Owen and Veasey, 2020). Sleep disruption decreases the number of neurons in the locus coeruleus (Zhang et al., 2014), hypothalamus (Zhu et al., 2016), amygdala (Zhu et al., 2018), and prefrontal cortex (Noorafshan et al., 2017). The progressive loss of functional neurons following disturbed sleep results in neurobehavioral impairments, and together this neuronal loss and behavioral phenotype is defined as neurodegeneration (Owen and Veasey, 2020).

The aberrant accumulation and distribution of Aβ, tau, alpha synuclein (α-syn), and TarDNA binding protein 43 (TDP-43) are common pathological features of neurodegenerative diseases, and this aberrant protein aggregation is exacerbated by sleep abnormalities (Owen and Veasey, 2020). Whereas the presence of fibrillar protein aggregates, such as amyloid plaques (Aβ), neurofibrillary tangles (tau), and Lewy bodies (α-syn) are defining pathological features of individual neurodegenerative diseases, they may not be the earliest markers. Rather, generation of small soluble toxic oligomeric variants of these pathological proteins likely precedes the formation of the hallmark fibrillar aggregates.

We previously developed single chain antibody variable domain (scFv) based reagents that selectively bind disease-related variants of tau, Aβ, TDP-43, and α-syn. These small oligomeric variants are present in post-mortem human neurodegenerative disease brain tissue, but not in cognitively normal, age-matched human samples (Emadi et al., 2007, 2009; Zameer et al., 2008; Kasturirangan et al., 2010; Tian et al., 2015; Williams et al., 2015b). This panel of reagents can be used to identify protein variant fingerprints that correlate with different neurodegenerative diseases (Williams et al., 2015a,b, 2017a,b; Venkataraman et al., 2020a,b), and can detect blood-based biomarkers during early, presymptomatic stages of AD (Cho et al., 2022). We have also shown that these same protein variants are detected in human brain after chronic traumatic brain injury (TBI), and that respective protein variant fingerprints of blood samples taken from individual TBI cases mirror neurodegenerative disease-specific protein variant fingerprint signatures (Williams et al., 2018). Our preclinical TBI studies in wild-type mice demonstrate that experimental diffuse TBI increases toxic protein variants that correspond to brain injury-induced behavioral deficits (Panayi et al., 2024).

Chronic sleep loss alters amyloid processing, tau and tauopathy, and α-syn aggregation (Owen and Veasey, 2020). The extent to which, and how acute sleep loss alters protein pathology is less clear. Furthermore, it is not known if specific brain regions are differentially susceptible to sleep loss-induced neurodegenerative pathologies. In the current study, we subjected wild-type mice to 6 hours of sleep deprivation for five consecutive days and measured toxic protein variants in six brain regions of interest. We hypothesized that acute sleep deprivation would lead to accumulation of toxic oligomeric variants of Aβ, tau, α-syn, and TDP-43 in a region-specific manner. These studies explore the mechanistic link between protein pathology resulting from sleep deprivation and the hallmark pathology of neurodegenerative diseases, such as Alzheimer’s disease.

## 2 Materials and methods

### 2.1 Rigor

All animal studies were conducted in accordance with the guidelines established by the internal Institutional Animal Care and Use Committee (IACUC) and the National Institutes of Health (NIH) guidelines for the care and use of laboratory animals. Studies are reported following the Animal Research: Reporting *In Vivo* Experiments (ARRIVE) guidelines (Kilkenny et al., 2010). Randomization of animals was achieved by assigning mice to a manipulation group prior to initiation of the study to ensure equal distribution among groups. Data collection stopped at pre-determined final endpoints based on days post-sleep deprivation. All histology was scored by investigators blinded to the treatment groups.

### 2.2 Animals

Adult male (10–12 weeks old) C57BL/6 mice (Envigo/Inotiv; C57BL/6NHsd) were used for all experiments (*n* = 10). Mice were housed in a 14 h light:10 h dark cycle at a constant temperature (23°C ± 2°C) with food and water available *ad libitum*. Mice were evaluated daily during the sleep deprivation protocol via physical examination of each animal’s condition. Animal care was approved by the Institutional Animal Care and Use Committee at the University of Arizona (13-460).

### 2.3 Sleep deprivation

Mice were randomly assigned to a sleep deprivation or no sleep deprivation (control) group. Control mice were group housed in a standard shoebox cage and remained undisturbed throughout the experimental period. Mice in the sleep deprivation group were group housed in a standard shoe box cage but were removed daily and placed in an open top cage, as a group, where they received continuous sleep deprivation for 6 h a day [zeitgeber (ZT) 0–6], for five consecutive days. Sleep deprivation was accomplished using a minimally stressful gentle handling method, as we have previously published (Rowe et al., 2014). For sleep deprivation, mice were under constant observation and the cage was tapped or the mouse was gently touched with a cotton applicator stick when visible signs of sleep were present (Patti et al., 2010).

### 2.4 Tissue preparation

Immediately following sleep deprivation on day 5, brain tissue was collected from all mice. Mice were injected intraperitoneally with Euthasol and perfused with iced 1× phosphate buffered saline (PBS). Brains were removed and post-fixed in 4% paraformaldehyde (PFA) for 24 h. Brains were cryoprotected in successive concentrations of sucrose (15, 30%). Fixed brains were cryosectioned in the coronal plane through regions of interest at 40 μm and were mounted on slides and stored at −80°C.

### 2.5 scFv-phage production

**Figure 1:**
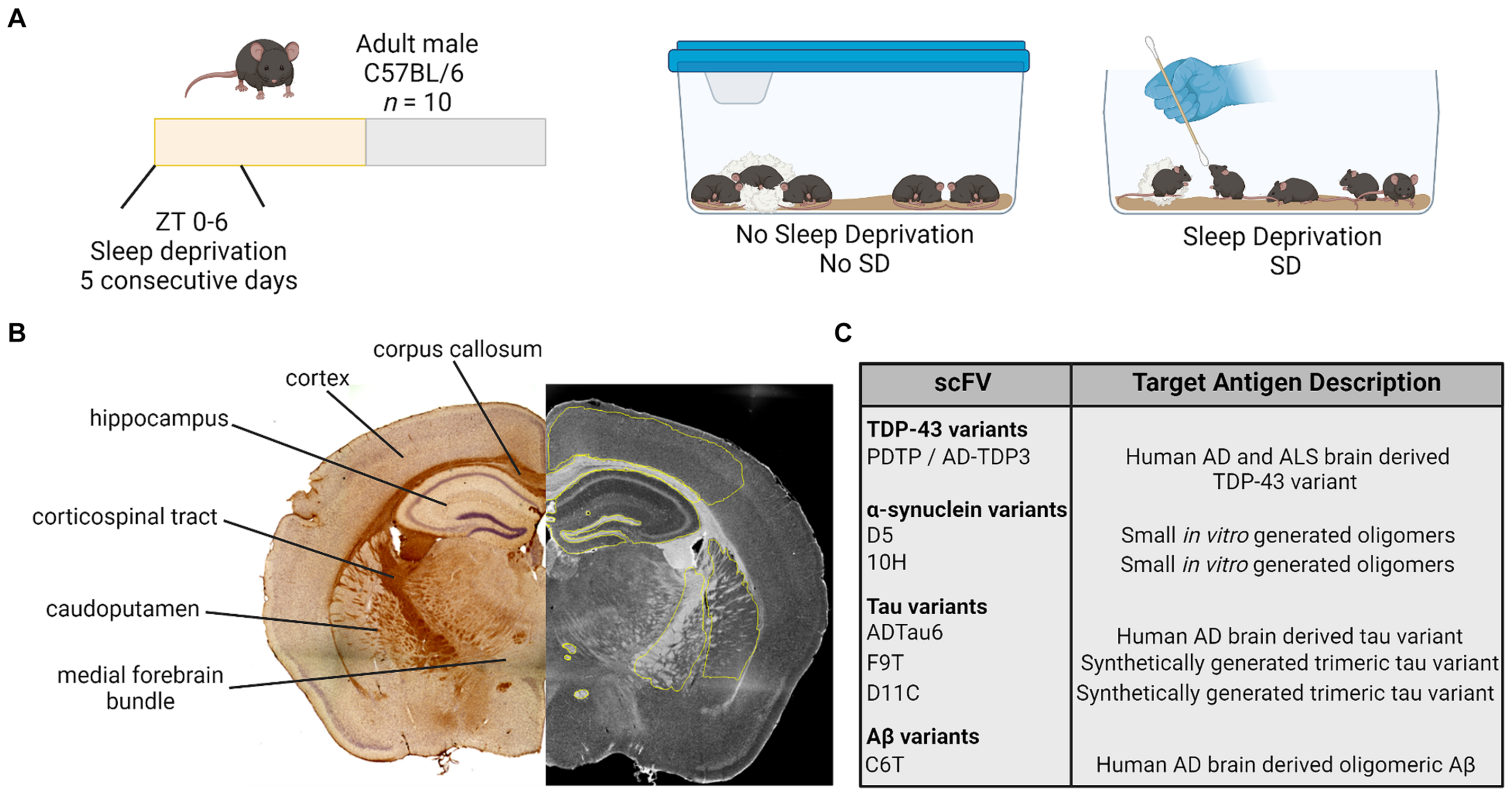
Study design. **(A)** Adult male mice were randomly assigned to a manipulation and received no sleep deprivation (undisturbed sleep) or were manually sleep deprived using a cotton applicator stick for the first 6 h of the light period [Zeitgeber (ZT) 0–6] for 5 consecutive days. **(B)** On day 5, brain tissue was collected and processed for immunohistochemistry. Antibody staining intensity was quantified in six regions of interest. **(C)** Brain tissue was stained for variants of TDP-43, α-synuclein, tau, and amyloid β (Aβ) using a panel of seven scFvs. AD, Alzheimer’s disease; ALS, amyotrophic lateral sclerosis; TDP-43, TAR DNA binding protein 43; scFvs, single chain antibody variable domains.

Seven different scFvs targeting the protein variants of interest were utilized (Figure 1) (Emadi et al., 2007, 2009; Zameer et al., 2008; Kasturirangan et al., 2010; Tian et al., 2015). Each scFv was expressed as previously described (Williams et al., 2015b). Briefly, the expression plasmids encode the scFv connected to the M13 phage minor coat protein pIII, for expression of the scFv on the phage surface. TG1 cells containing the plasmids were incubated with M13 hyperphage (Progen, Germany) or KM13 helper phage overnight to generate the scFv-phage fusion construct. Scfv fusion phage were purified by repeated polyethylene glycol (PEG 8000) precipitation and centrifugation. See Supplementary Table S1 for a list of scFvs, their target antigens, and validated targets in the literature.

### 2.6 Chromogenic DAB immunostaining

Brain tissue was stained for the presence of Aβ, tau, TDP-43, and α-syn variants utilizing the scFv panel (Emadi et al., 2007, 2009; Zameer et al., 2008; Kasturirangan et al., 2010; Tian et al., 2015). Serial brain sections of six brain regions from each mouse were separately probed with each scFv (Figure 1).

Immunostaining was performed as described previously (He et al., 2019, 2021). Tissue sections underwent a 10 min-high-temperature antigen retrieval step and incubated with scFv-phage (1:1,000) for 1 h; then placed in anti-M13 mouse antibody (Invitrogen 1:4,000) incubation for 1 h. Sections were washed and incubated with biotinylated anti-mouse IgG horse antibody (1:1,000). Following washing, the Vectastain ABC-HRP kit (Vector Laboratories, Burlingame, CA) was applied. Samples were visualized using 3,3′-diaminobenzidine (DAB) as a substrate (Vector Laboratories). Sections were counterstained with hematoxylin.

### 2.7 Immunostaining image analysis

Brightfield images were collected at 4× using a Keyence BZ-X810 and a Leica TCS SP5 LSCM or EVOS M7000 in the Regenerative Medicine Imaging Facility at Arizona State University. The 4× image stitching was performed using Keyence software or NIH Image J 1.52p (FIJI 2). Antibody staining was measured using Keyence Analyzer software or NIH ImageJ with a measured mean grey value intensity (integrated densitometry/area) of phage staining ranging from 0–255, (0 = no stain; 255 = hypothetical high) for selected regions of interest. Regions of interest from which antibody staining was measured included (Figure 1): cortex; corpus callosum; hippocampus; corticospinal tract; caudoputamen; and medial forebrain bundle with focus on the columns of the fornix and mammillothalamic tract. One to two sections were imaged and analyzed per mouse, and these values were pooled for a single value per hemisphere. Each hemisphere was measured separately and values from each hemisphere are presented as individual raw data points for each mouse. Care was taken when analyzing tissue to avoid anomalous regions where tissue had tears or wrinkles, or areas where tissue overlapped. See Supplementary Figure S1 for representative images.

### 2.8 Statistical analysis

Statistical analyses were performed in GraphPad Prism 9.5.1 (San Diego, California United States) software. Values for each antibody staining image were analyzed as raw values for each hemisphere. An unpaired t-test was used to determine statistical differences (*p* < 0.05) in staining intensity of tissue from sleep deprived mice vs. control mice.

## 3 Results

### 3.1 Sleep deprivation variably induced expression of small toxic protein variants in the brain regions of interest assayed in this study

#### 3.1.1 TDP-43 variant

**Figure 2:**
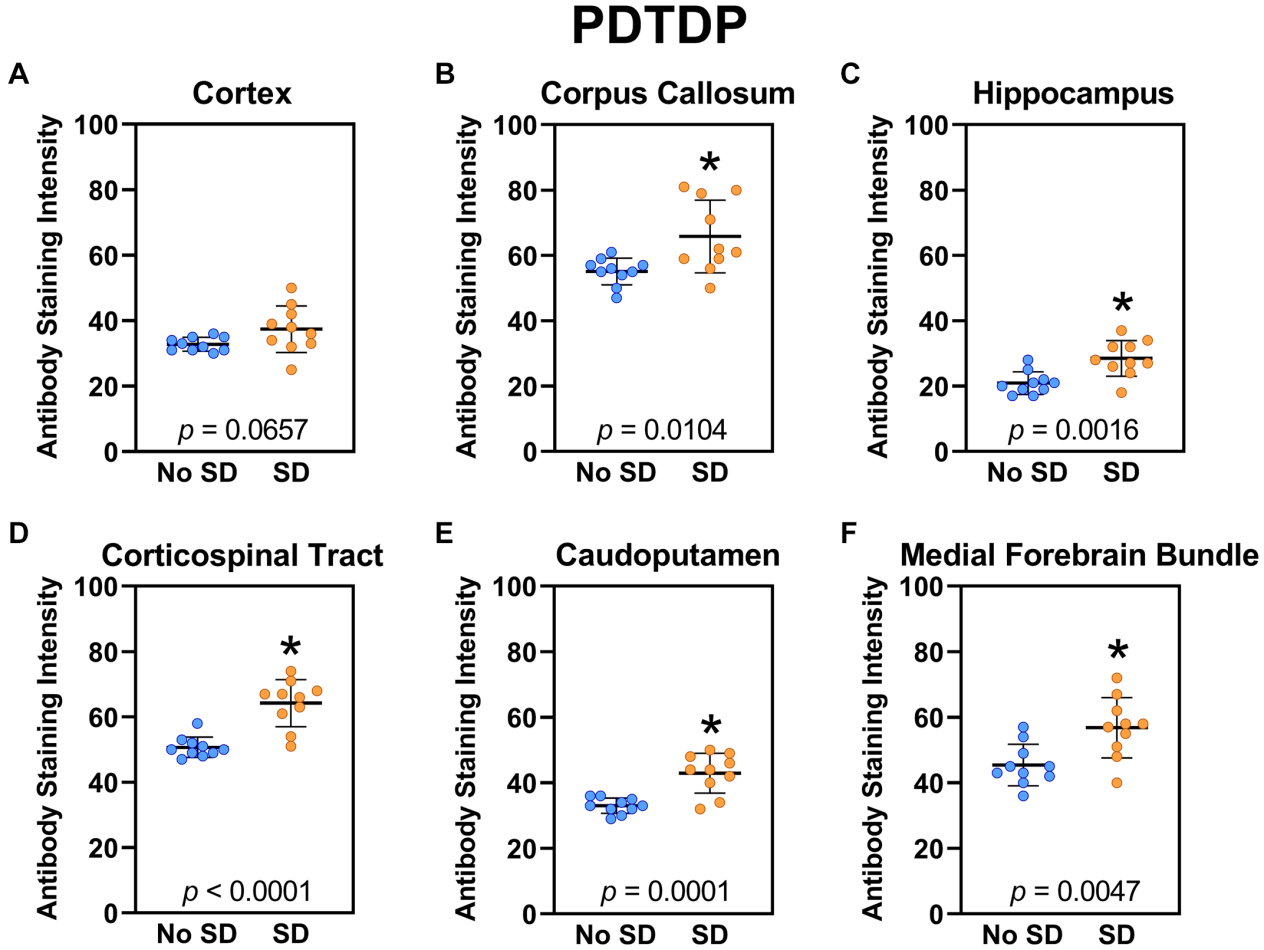
Sleep deprivation elevated brain wide levels of the TDP-43 variant targeted by PDTDP. **(A-F)** Expression of the TDP-43 variant was quantified across brain regions of interest. Mice subjected to sleep deprivation (SD) had significantly elevated levels of the TDP-43 variant in all regions of interest except the cortex, compared to mice with undisturbed sleep (No SD). Asterisks indicate *p* < 0.05 significance compared to No SD. Individual data points from each hemisphere are represented as colored dots. Error bars are ± SEM.

Sleep deprivation increased expression of the TDP-43 variant targeted by the PDTDP scFv in the corpus callosum (*t*_18_ = 1.96, *p* = 0.0104), hippocampus (*t*_18_ = 3.72, *p* = 0.0016), corticospinal tract (*t*_18_ = 5.44, *p* < 0.0001), caudoputamen (*t*_18_ = 4.8, *p* = 0.0001), and the axons of the medial forebrain bundle (*t*_18_ = 3.23, *p* = 0.0047; Figure 2). There was increased expression of this TDP-43 variant in cortex (*t*_18_ = 1.96, *p* = 0.0657), but this failed to reach significance.

#### 3.1.2 Alpha synuclein variants

**Figure 3:**
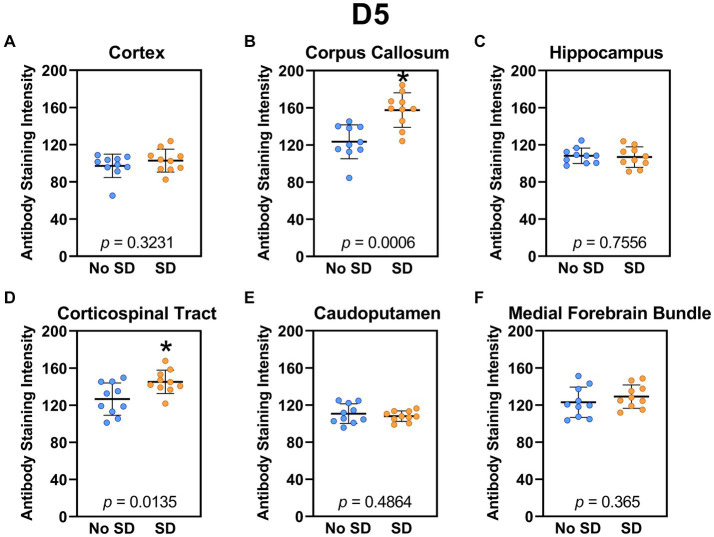
Sleep deprivation elevated levels of the α-synuclein variant targeted by D5 scFv. **(A-F)** The targeted a-syn variant expression was quantified across brain regions of interest. Mice subjected to sleep deprivation (SD) had significantly elevated levels of the D5 reactive a-syn variant in the **(B)** corpus callosum and **(D)** corticospinal tract compared to mice with undisturbed sleep (No SD). Asterisks indicate *p* < 0.05 significance compared to No SD. Individual data points from each hemisphere are represented as colored dots. Error bars are ± SEM.

**Figure 4:**
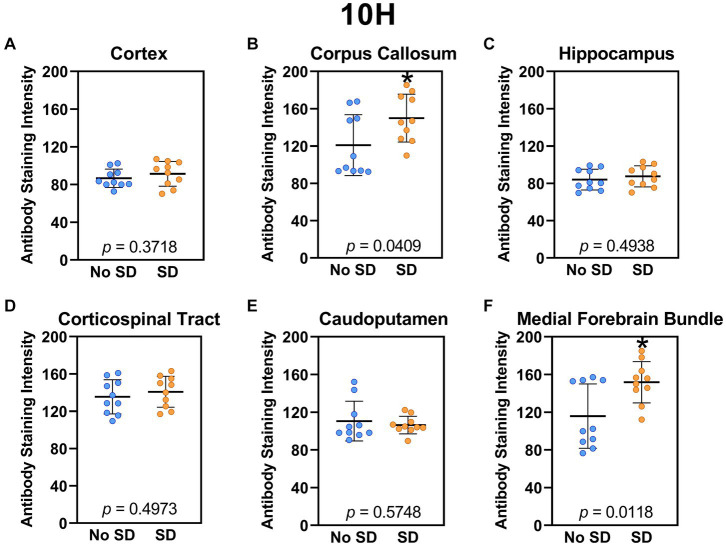
Sleep deprivation elevated levels of the α-synuclein variant targeted by the 10H scFv. **(A-F)** The targeted a-syn variant expression was quantified across brain regions of interest. Mice subjected to sleep deprivation (SD) had significantly elevated levels of the 10H reactive a-syn variant in the **(B)** corpus callosum and **(F)** axons of the medial forebrain bundle compared to mice with undisturbed sleep (No SD). Asterisks indicate *p* < 0.05 significance compared to No SD. Individual data points from each hemisphere are represented as colored dots. Error bars are ± SEM.

Sleep deprivation induced generation of a-syn variants targeted by the D5 (Figure 3) and 10H (Figure 4) scFvs in a region-specific manner. Sleep deprivation elevated D5 reactive a-syn only in the corpus callosum (*t*_18_ = 4.14, *p* = 0.0006) and corticospinal tract (*t*_18_ = 2.74, *p* = 0.0135), whereas 10H reactive a-syn was expressed in the corpus callosum (*t*_18_ = 2.2, *p* = 0.0409) and axons of the medial forebrain bundle (*t*_18_ = 2.8, *p* = 0.0118). There were no sleep deprivation-induced differences in D5 or 10H reactive a-syn variants in any of the other brain regions of interest.

#### 3.1.3 Tau variants

**Figure 5:**
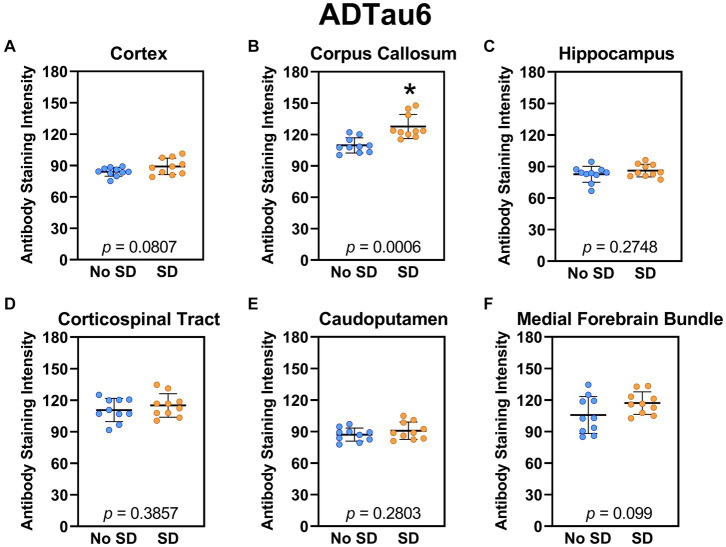
Sleep deprivation elevated levels of the tau variant targeted by the ADTau6 scFv in the corpus callosum. **(A-F)** The targeted tau variant expression was quantified across brain regions of interest. Mice subjected to sleep deprivation (SD) had significantly elevated levels of the tau variant targeted by ADTau6 in the **(B)** corpus callosum compared to mice with undisturbed sleep (No SD). Asterisks indicate *p* < 0.05 significance compared to No SD. Individual data points from each hemisphere are represented as colored dots. Error bars are ± SEM.

**Figure 6:**
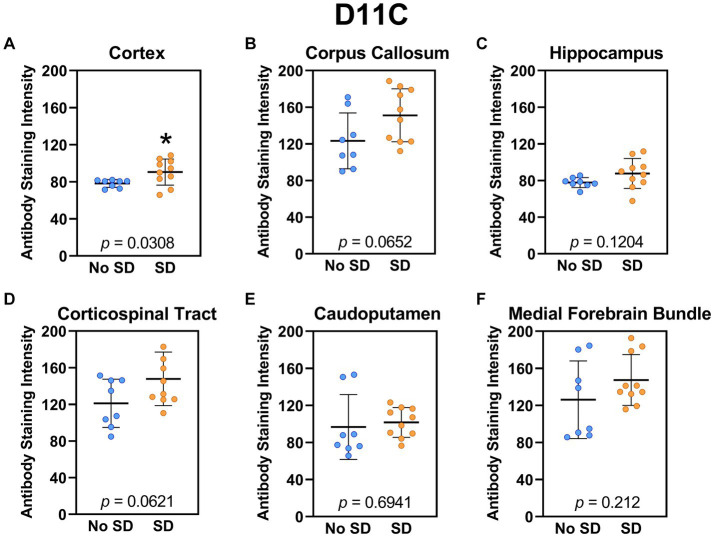
Sleep deprivation elevated levels of the tau variants targeted by the D11C scFv. **(A-F)** The targeted tau variant expression was quantified across brain regions of interest. Mice subjected to sleep deprivation (SD) had significantly elevated levels of the tau variant targeted by D11C in the **(A)** cortex compared to mice with undisturbed sleep (No SD). Asterisks indicate *p* < 0.05 significance compared to No SD. Individual data points from each hemisphere are represented as colored dots. Error bars are ± SEM.

**Figure 7:**
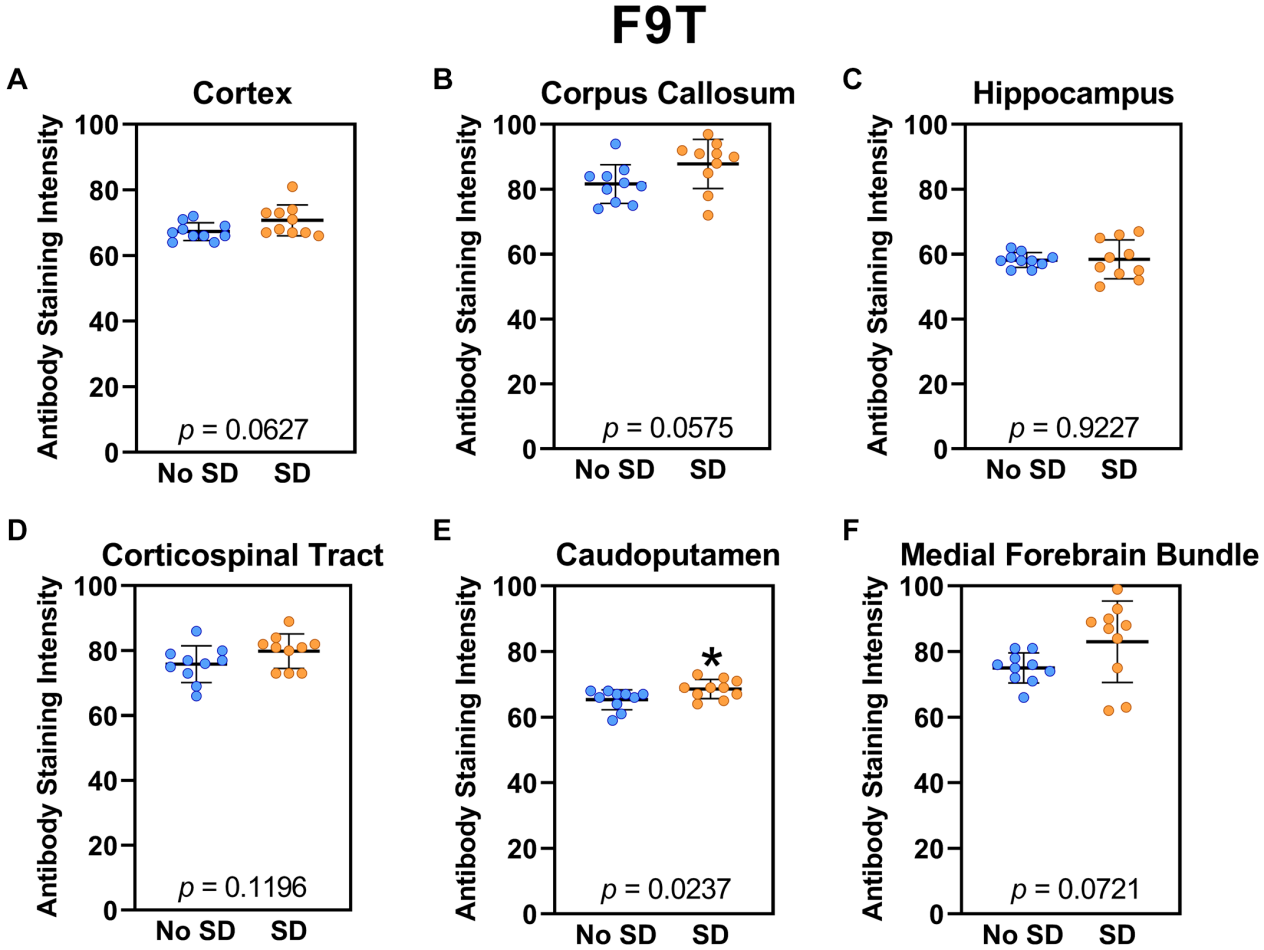
Sleep deprivation elevated levels of the tau variant targeted by the F9T scFv in the caudoputamen. **(A-F)** The targeted tau variant expression was quantified across brain regions of interest. Mice subjected to sleep deprivation (SD) had significantly elevated levels of the tau variant targeted by F9T in the **(E)** caudoputamen compared to mice with undisturbed sleep (No SD). Asterisks indicate *p* < 0.05 significance compared to No SD. Individual data points from each hemisphere are represented as colored dots. Error bars are ± SEM.

The tau variant targeted by the ADTau6 scFv was expressed only in the corpus callosum after sleep deprivation (*t*_18_ = 4.2, *p* = 0.0006; Figure 5). Sleep deprivation elevated levels of the tau variant targeted by the D11C scFv in the cortex (*t*_18_ = 2.369, *p* = 0.0308; Figure 6) and by the F9T scFv in the caudoputamen (*t*_18_ = 2.471, *p* = 0.0237; Figure 7). There was increased expression of the F9T targeted tau variant in the cortex (*t*_18_ = 1.98, *p* = 0.0627) and the corpus callosum (*t*_18_ = 2.03, *p* = 0.0575) but these failed to reach statistical significance. There were no sleep deprivation-induced differences in levels of the tau variants targeted by ADTau6 or F9T scFvs in other brain regions of interest.

#### 3.1.4 Amyloid beta variant

**Figure 8:**
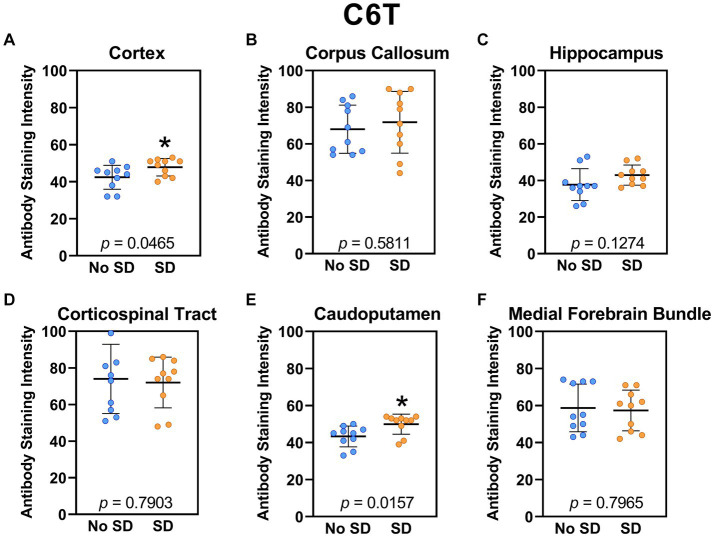
Sleep deprivation elevated levels of the amyloid-beta variant targeted by the C6T scFv. **(A-F)** The targeted amyloid-beta variant expression was quantified across brain regions of interest. Mice subjected to sleep deprivation (SD) had significantly elevated levels of the tau variant targeted by C6T in the **(A)** cortex and **(E)** caudoputamen compared to mice with undisturbed sleep (No SD). Asterisks indicate *p* < 0.05 significance compared to No SD. Individual data points from each hemisphere are represented as colored dots. Error bars are ± SEM.

Sleep deprivation elevated levels of the Aβ variant targeted by the C6T scFv only in the cortex (*t*_18_ = 2.14, *p* = 0.0465) and caudoputamen (*t*_18_ = 2.67 *p* = 0.0157; Figure 8).

## 4 Discussion

Co-morbid sleep disturbances are common in patients with neurological disorders such as amyotrophic lateral sclerosis (ALS) (Ahmed et al., 2015), dementia with Lewy bodies (DLB) (Elder et al., 2022), frontotemporal disorders/dementia (FTD) (Sani et al., 2019), Parkinson’s disease (PD) (French and Muthusamy, 2016), and Alzheimer’s disease (AD) (Vitiello and Borson, 2001; Urrestarazu and Iriarte, 2016; Vanderheyden et al., 2018). Data also suggest that sleep disturbances may precede the onset of neurological disorders. A meta-analysis of longitudinal studies that included 246,786 participants at baseline, and 25,847 dementia cases, concluded that sleep disturbances may predict the risk of incident of dementia (Shi et al., 2018). Because disturbed sleep is a strong risk factor for developing dementia and other neurodegenerative diseases, it is important to understand how sleep loss contributes to these pathologies. Identifying the type and location of specific pathologies induced by sleep loss may elucidate the mechanistic link between sleep disturbances/disorders and neurodegenerative diseases, and potentially guide therapeutic interventions.

Clinical and pathological subtypes of ALS and FTD are characterized by TDP-43 immunoreactivity (Davidson et al., 2007; Tan et al., 2007). In this study we found that a variant of TDP-43 derived from human ALS brain tissue and targeted by PDTDP scFv, is expressed throughout the brain after sleep deprivation. TDP-43 is a highly conserved and ubiquitously expressed protein in brain tissue (Buratti et al., 2001) that is typically localized to the nucleus of neurons and glial cells (Mackenzie and Rademakers, 2008). The abnormal aggregation of TDP-43 in neurons and glia is a pathological hallmark of ALS and FTD (Arseni et al., 2022). TDP-43 aggregation is also common in AD and PD (Arseni et al., 2022). Although pathological TDP-43 aggregates contain abnormal post-translational modifications (e.g., ubiquitylation, phosphorylation), there is limited understanding of their structures or the extent to which, and mechanisms by which TDP-43 contributes to neurodegenerative diseases (Arai et al., 2006). Sleep impairment may increase neuronal stress and disrupt TDP-43 homeostasis (Morrone et al., 2023), which could lead to abnormal aggregations. In the current study, we found that sleep deprivation increased expression of the TDP-43 variant targeted by PDTDP in all brain regions assayed, except the cortex. Sleep loss alters the ubiquitin proteasome system and can affect protein phosphorylation (Morrone et al., 2023). Sleep deprivation of mice differentially phosphorylates proteins. Thus, it is plausible that sleep deprivation in our study induced post-translational modifications to TDP-43 that resulted in the formation of pathological variants (Xu et al., 2022).

In addition to TDP-43 variants, sleep deprivation increases expression of small oligomeric a-syn variants targeted by the D5 and 10H scFvs. α-syn, a presynaptic neuronal protein, is neuropathologically linked to Parkinson’s disease and abnormal deposition occurs in early stages of the disease process (Stefanis, 2012). Aberrant soluble oligomeric conformations of α-syn (i.e., protofibrils) are toxic and mediate neuronal death through disrupted cellular homeostasis (Stefanis, 2012). In humans, sleep loss increases α-syn, which aggregates in the brains of individuals with diagnosed Parkinson’s disease (Holth et al., 2019). In a rodent model of Parkinson’s disease, sleep deprivation increases aggregates of α-syn; enhancing slow wave sleep reduces the burden of α-syn, potentially by increasing glymphatic function and modulating protein homeostasis (Morawska et al., 2021). Like TDP-43, α-syn undergoes post-translational modifications, such as phosphorylation. Thus, sleep deprivation may increase oligomerization, as evidenced by increased expression of the a-syn variants targeted by both D5 and 10H.

Tau pathologies occur in early stages of AD. Sleep deprivation in humans increases tau levels in cerebral spinal fluid (Holth et al., 2019), and preclinical data indicate that sleep restriction increases hyperphosphorylation of tau proteins (Rothman et al., 2013). Sleep deprivation of mice in this study increases expression of the tau variant targeted by the ADTau6 scFv, a tau variant derived from human AD brain, and tau variants targeted by F9T and D11C, both of which are synthetically generated trimeric tau variants. Whereas tau aggregates into fibrils and higher order neurofibrillary tangles, soluble oligomeric tau species may play a critical role in AD progression because their expression correlates with neuronal loss and cognitive function (Tian et al., 2013). We previously reported that trimeric, but not monomeric or dimeric tau oligomers, were neurotoxic at low nanomolar concentrations (Tian et al., 2013). Our data demonstrate that sleep deprivation increases trimeric and other soluble tau variants in mouse brain, suggesting a potential mechanism by which sleep deprivation may drive the etiology AD pathology. Sleep deprivation may also promote AD pathogenesis by facilitating the spread of tau through synaptically-linked networks (Holth et al., 2019; Barthelemy et al., 2020).

In addition to tau neurofibrillary tangles, AD pathogenesis is also characterized by deposition of Aβ (Murphy and LeVine, 2010). Cerebral spinal fluid concentrations of Aβ increase by 30% following a night of sleep deprivation, suggesting that disrupted sleep may increase AD risk by increased production or decreased clearance of Aβ (Lucey et al., 2018). Sleep deprivation of mice increases expression of the Aβ variant targeted by the C6T scFv, an oligomeric Aβ variant derived from human AD brain tissue. We hypothesize that sleep deprivation increases C6T immunoreactivity because of increased production and decreased clearance of the Aβ variant (Shokri-Kojori et al., 2018). It is important to note the bidirectional relationship between sleep and AD. While disturbed sleep increases AD pathology, such as concentrations of Aβ, AD itself also disturbs sleep in patients (Cordone et al., 2019). Sleep disturbances are one of the leading causes of patient institutionalization for individuals suffering from AD (Moran et al., 2005).

Although sleep deprivation increases immunoreactivity for all of the neurodegenerative disease associated oligomeric variants assayed in this study, their localization is generally region-specific; only the TDP-43 variant targeted by PDTDP is expressed in each of the brain regions of interest. Immunoreactivity for four of the seven variants assayed increased in the corpus callosum (PDTDP, D5, 10H, ADTau6). A recent meta-analysis of neuroimaging studies identified the corpus callosum as a brain region affected by sleep deprivation, suggesting that it may be a neuroanatomic substrate contributing to brain damage induced by sleep deprivation (Zhang et al., 2024). Of the small toxic proteins assayed in this study, only PDTDP immunoreactivity increases in the hippocampus, suggesting that this brain region is not vulnerable to neuropathological damage by other oligomeric variants after sleep deprivation. Sleep deprivation impairs hippocampal synaptic plasticity, hippocampus-dependent memory, and learning (Prince and Abel, 2013; Havekes and Abel, 2017). In mice, sleep deprivation increases tau and Aβ in the hippocampal extracellular space (Roh et al., 2012; Holth et al., 2019). As such, we expected to detect immunoreactivity of toxic tau and Aβ oligomers in this brain region. Additional research is necessary to characterize other brain regions of interest to understand the implications of regional specificity for immunoreactivity of these toxic protein variants.

As with all studies, there are some limitations to this one. We used only male mice, and additional studies are needed to determine if sex is a relevant biological variable for elucidating the pathological response to sleep deprivation under the conditions our study. Studies using female mice are important because, compared to men, roughly twice as many women have AD. The duration (acute or chronic) of sleep deprivation affects cellular responses. Age of the mice (10–12 weeks old) could also contribute to our findings. It is likely that sleep deprivation in middle-aged or aged mice would result in a considerable increase in the amount of neuropathology, and sleep deprivation experiments in older mice are warranted. In this study, mice had 18 h of recovery sleep opportunity between each sleep deprivation period. Increased sleep during the recovery periods between sleep deprivation may have facilitated clearance of toxic protein variants and as such, reduced their accumulation. A protocol in which longer periods of chronic sleep deprivation are used may exacerbate neurodegenerative pathology due to increased toxic oligomer production and reduced clearance. In addition, sleep disruption may be more clinically relevant than total sleep deprivation and a protocol in which sleep is chronically disrupted may yield different results. In this study we focused on the impact of sleep deprivation on the accumulation of seven toxic neurodegenerative disease associated protein variants in brain regions implicated in pathologies associated with these diseases. Future research could determine the impact on sleep of expression of these and other protein variants in other brain regions. It should also be noted that mice in this study were housed in a 14 h light:10 h dark cycle, which is standard for the University of Arizona—Phoenix vivarium, and common with commercial and vendor facilities. However, this extended light period when mice typically sleep, could have allowed for additional rebound sleep following daily sleep deprivation.

In conclusion, sleep disturbances often precede neurodegenerative diseases, and small soluble toxic oligomeric variants of pathological proteins may not only drive pathology, but importantly may potentially be biomarkers for detection of neurodegeneration. In this study, we demonstrated that acute sleep deprivation of wild type mice increases immunoreactivity of toxic variants of TDP-43, α-syn, tau, and Aβ. These data provide a mechanistic link between protein pathology resulting from sleep deprivation and the hallmark pathology of neurodegenerative diseases, such as Alzheimer’s disease and Parkinson’s disease.

## Data availability statement

The raw data supporting the conclusions of this article will be made available by the authors, without undue reservation.

## Ethics statement

The animal study was approved by University of Arizona Institutional Animal Care and Use Committee. The study was conducted in accordance with the local legislation and institutional requirements.

## Author contributions

RR: Conceptualization, Funding acquisition, Supervision, Visualization, Writing – original draft, Writing – review & editing. PS: Data curation, Formal analysis, Methodology, Writing – review & editing. PH: Formal analysis, Methodology, Writing – review & editing. GM: Formal analysis, Visualization, Writing – review & editing. MO: Supervision, Writing – review & editing. MS: Conceptualization, Funding acquisition, Methodology, Resources, Supervision, Writing – review & editing.
