# Supplementary material for: Acute sleep deprivation in mice generates protein pathology consistent with neurodegenerative diseases
Source: Front Neurosci. 2024 Jul 24;18:1436966. doi: 10.3389/fnins.2024.1436966 (PMC11303328; doi:10.3389/fnins.2024.1436966)
Supplement: Supplementary file 1 [file Data_Sheet_1.docx]

| **Supplementary Table 1:** List of scFvs and their target antigens | | | |
| --- | --- | --- | --- |
| scFv | | Target Antigen Description | Validated targets |
| **Tau variants** | | | |
| F9T | | Synthetically generated trimeric tau variant | Human AD brain tissue, blood samples, neuronal cell culture, AD mouse model, human TBI blood samples [1; 2; 3] |
| D11C | | Synthetically generated trimeric tau variant | Human AD brain tissue, blood samples, neuronal cell culture, AD mouse model, human TBI blood samples [1; 2; 3; 4] |
| ADTau6 | | Human AD brain derived tau variant | Human AD brain tissue, blood samples [5] |
| **Aβ variants** | | | |
| C6T | | Human AD brain derived oligomeric Aβ | Human AD brain tissue, blood samples, human TBI blood samples, AD mouse model [1; 3; 4; 6; 7; 8] |
| **α-synuclein variants** | | | |
| 10H | Small *in vitro* generated oligomers | | Human PD brain tissue, blood samples, human TBI blood samples [8; 9; 10; 11] |
| D5 | | Small *in vitro* generated oligomers | Human PD brain tissue, blood samples, human TBI blood samples[8; 9; 10; 11] |
| **TDP-43 variants** | | | |
| PDTDP | | Human PD and ALS brain derived TDP-43 variant | Human PD brain tissue, blood samples [8; 12] |


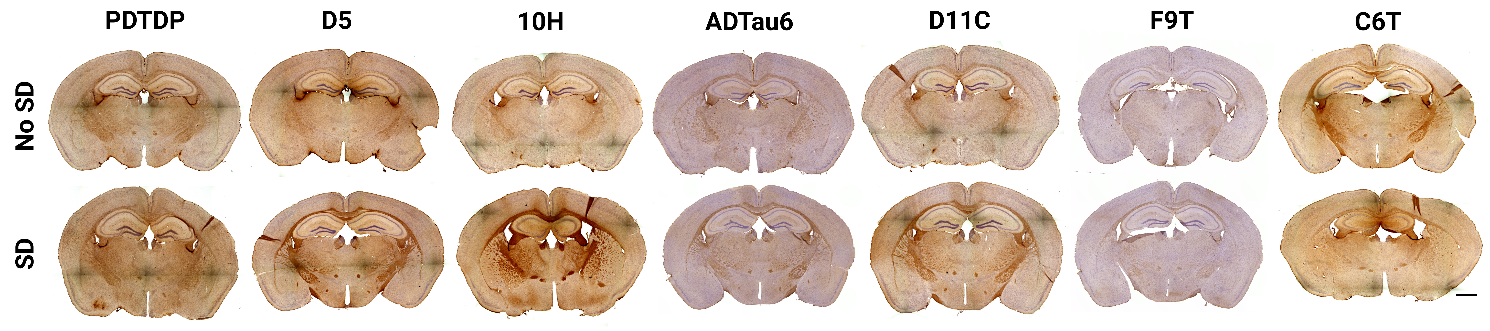


**Supplementary Figure 1:** Representative images of tissue from mice subjected to sleep deprivation (SD) or no sleep deprivation (No SD). Tissue was stained for the TDP-43 variant targeted by PDTDP; α-syn variant targeted by D5 and 10H; tau variant targeted by ADTau6, D11C, and F9T; and amyloid-beta variant targeted by C6T. Scale bar = 1mm.

**References:**

[1] S.M. Williams, C. Peltz, K. Yaffe, P. Schulz, and M.R. Sierks, CNS disease-related protein variants as blood-based biomarkers in traumatic brain injury. Neurology 91 (2018) 702-709.

[2] H. Tian, E. Davidowitz, P. Lopez, S. Emadi, J. Moe, and M. Sierks, Trimeric tau is toxic to human neuronal cells at low nanomolar concentrations. International journal of cell biology 2013 (2013) 260787.

[3] H.J. Cho, P. Schulz, L. Venkataraman, R.J. Caselli, and M.R. Sierks, Sex-Specific Multiparameter Blood Test for the Early Diagnosis of Alzheimer's Disease. International journal of molecular sciences 23 (2022).

[4] S.M. Williams, P. Schulz, T.L. Rosenberry, R.J. Caselli, and M.R. Sierks, Blood-Based Oligomeric and Other Protein Variant Biomarkers to Facilitate Pre-Symptomatic Diagnosis and Staging of Alzheimer's Disease. J Alzheimers Dis (2017).

[5] L. Venkataraman, P. He, P. Schulz, and M.R. Sierks, Isolation and characterization of antibody fragment selective for human Alzheimer’s disease brain-derived tau variants. Neurobiology of Aging 94 (2020) 7-14.

[6] P. He, P. Schulz, and M.R. Sierks, A conformation specific antibody against Oligomeric β-Amyloid Restores Neuronal Integrity in a Mouse Model of Alzheimer's Disease. J Biol Chem (2021) 100241.

[7] S. Kasturirangan, T. Reasoner, P. Schulz, S. Boddapati, S. Emadi, J. Valla, and M.R. Sierks, Isolation and characterization of antibody fragments selective for specific protein morphologies from nanogram antigen samples. Biotechnol Prog 29 (2013) 463-71.

[8] S.M. Williams, P. Schulz, and M.R. Sierks, Oligomeric alpha-synuclein and beta-amyloid variants as potential biomarkers for Parkinson's and Alzheimer's diseases. The European journal of neuroscience 43 (2016) 3-16.

[9] S. Emadi, S. Kasturirangan, M.S. Wang, P. Schulz, and M.R. Sierks, Detecting morphologically distinct oligomeric forms of alpha-synuclein. J Biol Chem 284 (2009) 11048-58.

[10] S. Emadi, H. Barkhordarian, M.S. Wang, P. Schulz, and M.R. Sierks, Isolation of a human single chain antibody fragment against oligomeric alpha-synuclein that inhibits aggregation and prevents alpha-synuclein-induced toxicity. J Mol Biol 368 (2007) 1132-44.

[11] W. Xin, S. Emadi, S. Williams, Q. Liu, P. Schulz, P. He, N.B. Alam, J. Wu, and M.R. Sierks, Toxic Oligomeric Alpha-Synuclein Variants Present in Human Parkinson's Disease Brains Are Differentially Generated in Mammalian Cell Models. Biomolecules 5 (2015) 1634-51.

[12] S.M. Williams, G. Khan, B.T. Harris, J. Ravits, and M.R. Sierks, TDP-43 protein variants as biomarkers in amyotrophic lateral sclerosis. BMC Neurosci 18 (2017) 20.
